# Distinct corneal morphometric remodeling in early keratoconus: Insights from sirius scheimpflug–placido tomography and quantitative epithelial pattern assessment–a retrospective cross-sectional study

**DOI:** 10.1097/MD.0000000000049687

**Published:** 2026-07-10

**Authors:** Hande Hüsniye Telek, Huri Sabur, Tugce Horozoglu Ceran, Yaprak Arzu Özdemir, Serkan Akkaya

**Affiliations:** a Department of Ophthalmology, University of Health Sciences Ankara Training and Research Hospital, Ankara, Turkey; b Department of Ophthalmology, Bolu Education and Research Hospital, Bolu, Turkey; c Department of Ophthalmology, Afyonkarahisar Health Sciences University, Afyonkarahisar, Turkey; d Department of Statistics, Gazi University Faculty of Science, Ankara, Turkey; e Department of Ophthalmology, University of Health Sciences Ankara Training and Research Hospital, Ankara, Turkey.

**Keywords:** corneal morphometric remodeling, early-stage keratoconus, higher-order aberrations, quantitative epithelial pattern assessment, sirius scheimpflug–placido tomography

## Abstract

This study aimed to investigate the diagnostic performance of Sirius Scheimpflug–Placido tomography parameters in distinguishing early-stage keratoconus (KC) from normal eyes and to determine whether quantitative epithelial pattern assessment adds incremental value to these measurements. In this retrospective cross-sectional study, 115 eyes were included: 80 eyes with early-stage KC (Stage 1, n = 38; Stage 2, n = 42) and 35 age-matched healthy controls. Only patients with unilateral KC were enrolled in the KC group, and the affected eye was selected for evaluation, whereas only the right eye of each participant was included in the control group. Corneal imaging was performed using the Sirius system to measure anterior and posterior curvature, pachymetry, corneal volume, anterior chamber depth, anterior chamber volume, KC-specific indices, and higher-order aberrations. Quantitative epithelial pattern assessment was conducted with anterior segment optical coherence tomography, evaluating the mean, minimum, maximum, and range of epithelial thickness within the central 6-mm zone. Anterior corneal curvature (Kmax, SimK1, SimK2) and pachymetric parameters (thinnest corneal thickness, corneal volume [Kvol]) significantly differed between keratoconic and control eyes (*P* < .001), with progressive changes from Stage 1 to Stage 2. Posterior corneal indices (Symmetry Index Back, Keratoconus Vertex Back, Baiocchi–Calossi–Versaci Back) showed pronounced differences, reflecting early ectatic changes. Higher-order aberrations, particularly vertical coma, increased with disease stage. Epithelial pattern assessment revealed focal thinning over the cone apex and increased range, correlating with posterior elevation and coma, highlighting subtle remodeling even in early KC. Sirius Scheimpflug–Placido tomography provides highly effective diagnostic metrics for identifying early KC, with quantitative epithelial pattern assessment serving as a supportive biomarker that refines diagnostic accuracy in ambiguous presentations. These findings highlight the clinical relevance of combining corneal morphometric and epithelial remodeling analyses for enhanced early detection of KC.

## 1. Introduction

Keratoconus (KC) represents a biomechanical and structural disorder of the cornea in which progressive thinning and asymmetric steepening lead to irregular astigmatism and visual distortion.^[1,2]^ Although the pathophysiological mechanisms underlying KC have been extensively studied, its earliest manifestations often remain elusive. By the time conventional topographic abnormalities emerge, subtle microstructural and optical changes have already taken place.^[3]^ Thus, the window for early detection when the disease is still amenable to stabilization remains critically narrow.

Traditional diagnostic methods, including keratometry and slit-lamp evaluation, lack the sensitivity to reveal these preclinical alterations.^[4]^ Over the past decade, technological advances in corneal imaging have enabled the transition from simple curvature mapping to comprehensive morphometric analysis. Among these, the Sirius Scheimpflug–Placido system integrates 2 imaging principles to generate a 3-dimensional reconstruction of the cornea and anterior segment. This hybrid platform not only provides high-resolution elevation data from both corneal surfaces but also quantifies pachymetric progression and higher-order aberrations (HOAs) with remarkable reproducibility.^[5–7]^ Such multidimensional data have made Sirius one of the most valuable diagnostic tools in differentiating subclinical KC from normal corneas.^[8,9]^

Yet, even advanced tomography may not fully capture the earliest stages of corneal ectasia. The corneal epithelium, capable of rapid adaptive remodeling, often masks or compensates for stromal irregularities.^[10]^ Quantitative analysis of epithelial patterns, rather than mere thickness mapping, can reveal subtle distribution asymmetries reflecting underlying biomechanical stress.^[11,12]^ When interpreted alongside Sirius-derived morphometric and aberrometric indices, these epithelial features may strengthen diagnostic confidence in eyes exhibiting borderline tomography.

In this context, we propose a comprehensive assessment model that integrates Sirius-based corneal morphometric profiling with quantitative epithelial thickness (ET) mapping (ETM) to refine early-stage KC detection. By correlating anterior and posterior corneal indices, pachymetric parameters, and epithelial distribution patterns, this study aims to define a distinctive diagnostic signature capable of distinguishing early ectatic changes from physiological variation with higher accuracy than single-modality approaches.^[13–15]^

## 2. Materials and methods

### 2.1. Study design and participants

This retrospective study was conducted at the Health Sciences University, Dişkapi Yildirim Beyazit Training and Research Hospital, after approval from the Dişkapi Scientific Research Ethics Committee (December 2022/Decision number: 145/16). Written informed consent was obtained from all participants. The study adhered to the Declaration of Helsinki. Data are available upon reasonable request.

Patients who visited the clinic between June 2021 and December 2022 with a confirmed diagnosis of KC based on retinoscopy, slit-lamp biomicroscopy, keratometry, and corneal tomography were included. Only Stage 1 and Stage 2 KC cases according to the Amsler–Krumeich classification were considered. Patients with bilateral KC, prior ocular surgery, corneal cross-linking (CXL), hydrops, or significant corneal scarring were excluded.

The control group consisted of age-matched healthy subjects (21–40 years) with no signs of KC on retinoscopy, biomicroscopy, or corneal tomography.

To avoid inter-eye correlation bias, only one eye per participant was included in the analysis. Accordingly, only patients with unilateral KC were enrolled in the KC group, and the affected eye was selected for evaluation. In the control group, only the right eye of each participant was included.

Sex distribution was additionally evaluated between groups to assess demographic comparability and potential confounding effects.

Exclusion criteria included prior refractive surgery, dry eye, systemic disease, spherical refraction > ±2.0 D, cylindrical refraction > ±2.0 D, mean keratometry > 47 D, or thinnest corneal thickness (TCT) < 475 μm.

### 2.2. Corneal imaging and quantitative epithelial pattern assessment

All participants underwent corneal tomography using a Sirius Scheimpflug–Placido system (Costruzione Strumenti Oftalmici, Florence, Italy) and epithelial evaluation using optical coherence tomography (AS-OCT). Imaging was performed between 9:00 and 12:00 am to reduce diurnal variation. Participants were instructed to fixate on the central light source while maintaining proper chin and forehead positioning.

Three high-quality scans were obtained for the selected eye using both devices, and the mean values were used for analysis. Only scans meeting predefined quality criteria (Sirius quality index ≥ 95%; AS-OCT signal strength ≥ 7/10) were included.

### 2.3. Measured parameters

#### 2.3.1. Corneal curvature and pachymetry

- Anterior corneal curvature: Kmax, SimK1, SimK2, and astigmatic axis.
- Pachymetric parameters: TCT and Kvol.
- Anterior chamber parameters: anterior chamber depth and anterior chamber volume.

#### 2.3.2. KC screening indices

- Anterior surface: Symmetry Index front (SIf), KC Vertex front (KVf), and Baiocchi–Calossi–Versaci front (BCVf).
- Posterior surface: Symmetry Index back (SIb), KC Vertex back (KVb), and Baiocchi–Calossi–Versaci back (BCVb).

### 2.4. Corneal aberrations

Total root mean square (RMS) and HOAs, including coma, trefoil, and spherical aberration, were measured over a 4-mm corneal zone.

### 2.5. Quantitative epithelial pattern assessment

Mean, minimum, maximum, and range (max–min) ET within the central 6-mm zone were evaluated. This metric reflects localized epithelial thinning and compensatory remodeling over the cone apex, providing sensitive indicators of subclinical ectatic changes.

### 2.6. Statistical analysis

Data were analyzed using IBM SPSS Statistics, version 23.0 (IBM Corp., Armonk), and R software, version 4.2.0 (R Foundation for Statistical Computing, Vienna, Austria). Continuous variables were expressed as mean ± standard deviation. Furthermore, to explicitly demonstrate the magnitude and precision of the observed effects, 95% confidence intervals for the mean differences were calculated for all pairwise group comparisons.

The control group was compared with Stage 1 and Stage 2, as well as Stage 1 and Stage 2 within the KC group for the examined variables. In these comparisons, the independent-samples Student *t*-test was applied for normally distributed data. A *P* value < .05 was considered statistically significant.

## 3. Results

### 3.1. Demographics and clinical characteristics

A detailed summary of the demographic and clinical characteristics of the participants is provided in Table 1. A total of 115 participants (115 eyes) were included in the study: 80 eyes with early-stage KC (Stage 1: 38 eyes, 19 females and 19 males; Stage 2: 42 eyes, 21 females and 21 males) and 35 age-matched healthy control eyes (17 females and 18 males). There was no significant difference in mean age among the groups (Control: 32.1 ± 4.5 years; Stage 1: 29.5 ± 4.2 years; Stage 2: 31.2 ± 6.1 years; *P* > .05).

**Table 1:** Group-wise mean, standard deviation, *P* values, and 95% CIs of mean differences for corneal curvature, TCT, anterior chamber parameters.

	Control (Mean ± SD)	Stage 1 (Mean ± SD)	Stage 2 (Mean ± SD)	*P* value* and CI (Control vs Stage 1)	*P* value* and CI (Control vs Stage 2)	*P* value* and CI (Stage 1 vs Stage 2)
Kmax (D)	44.16 ± 1.55	46.38 ± 1.43	50.25 ± 2.58	< .0001 [−2.91–−1.53]	< .0001 [−7.02–−5.16]	< .0001 [−4.77–−2.97]
SimK1 (D)	42.51 ± 1.46	42.63 ± 1.28	44.43 ± 1.48	.71 [−0.75–0.51]	< .0001 [−2.58–−1.26]	< .0001 [−2.40–−1.20]
SimK2 (D)	43.42 ± 1.62	45.22 ± 1.35	47.08 ± 1.44	< .0001 [−2.48–−1.12]	< .0001 [−4.35–−2.97]	< .0001 [−2.47–−1.25]
Astigmatic axis (°)	92.26 ± 61.64	92.64 ± 74.87	92.67 ± 65.48	.98 [−31.7–30.9]	.98 [−29.6–28.8]	.99 [−31.6–31.5]
TCT (µm)	530.89 ± 54.63	480.05 ± 36.09	476.02 ± 31.42	< .0001 [29.4–72.3]	< .0001 [34.4–75.3]	.5958 [−10.9–18.9]
Kvol (mm^3^)	56.78 ± 3.93	54.67 ± 3.37	53.53 ± 3.79	.0145 [0.43–3.79]	.0005 [1.53–4.97]	.1591 [−0.42–2.70]
ACV (mm^3^)	175.41 ± 29.35	189.24 ± 30.13	185.78 ± 35.40	.0484 [−27.5–−0.19]	.1751 [−24.8–4.09]	.6391 [−10.8–17.7]
ACD (mm)	3.05 ± 0.32	3.17 ± 0.31	3,23 ± 0,34	.1038 [−0.26–0.02]	.0214 [−0.32–−0.04]	.4120 [−0.20–0.08]

Astigmatic axis means the axis of the steepest corneal meridian (degrees), Kmax means the steepest curvature value of the cornea on the anterior tangential map (D), Kvol means corneal volume at 4-mm zone (mm^3^), SimK1 means the average sagittal corneal curvature between 4 to 8 Placido rings on the flattest meridian (D), SimK2 means the average sagittal curvature between 4 to 8 Placido rings on the steepest meridian (D), TCT means thinnest corneal thickness at 4-mm zone (µm).

ACD = anterior chamber depth (mm), ACV = anterior chamber volume (mm^3^), CI = condfidence interval, SD = standard deviation, TCT = thinnest corneal thickness.

* Student’s *t*-test. Values in square brackets represent the 95% confidence intervals of the mean differences.

Best-corrected visual acuity decreased progressively with disease stage (*P* < .001), while the magnitude of myopic shift and astigmatism increased correspondingly.

### 3.2. Corneal curvature parameters

Significant anterior corneal steepening was observed in keratoconic eyes. Kmax and SimK2 values increased from control (Kmax: 44.16 ± 1.55 D; SimK2: 43.42 ± 1.62 D) to Stage 1 (Kmax: 46.38 ± 1.43 D; SimK2: 45.22 ± 1.35 D) and Stage 2 (Kmax: 50.25 ± 2.58 D; SimK2: 47.08 ± 1.44 D), with all comparisons statistically significant (Table 1, *P* < .001). SimK1 showed a similar trend, while the astigmatic axis did not differ significantly among groups. These findings indicate progressive anterior corneal steepening as KC advances.

### 3.3. Pachymetric and anterior chamber parameters

TCT decreased significantly in Stage 1 (480.05 ± 36.09 µm) and Stage 2 (476.02 ± 31.42 µm) compared with controls (530.89 ± 54.63 µm, *P* < .001), whereas Kvol also declined progressively (Control: 56.78 ± 3.93 mm^3^; Stage 2: 53.53 ± 3.79 mm^3^; *P* < .001) (Table 1). anterior chamber depth and anterior chamber volume showed modest increases in KC, reflecting posterior corneal protrusion.

### 3.4. KC screening indices

Anterior surface indices (SIf, KVf, BCVf) demonstrated marked increases with disease stage, reflecting corneal asymmetry and ectatic deformation (Table 2). Posterior surface indices (SIb, KVb, BCVb) displayed even greater discriminatory power, with Stage 2 eyes exhibiting pronounced posterior elevation compared to Stage 1 and controls (Table 2). These results emphasize the diagnostic value of posterior corneal evaluation in early ectasia detection.

**Table 2:** Group-wise mean, standard deviation, *P* values and and 95% CIs of mean differences for keratoconus screening parameters from the corneal anterior surface and the corneal back surface.

	Control (Mean ± SD)	Stage 1 (Mean ± SD)	Stage 2 (Mean ± SD)	*P* value* and CI (Control vs Stage 1)	*P* value* and CI (Control vs Stage 2)	*P* value* and CI (Stage 1 vs Stage 2)
SIf (D)	0.28 ± 0.35	1.99 ± 0.93	3.45 ± 2.19	< .0001 [−2.03–−1.39]	< .0001 [−3.84–−2.50]	< .001 [−2.18–−0.74]
KVf (µm)	3.84 ± 1.14	9.75 ± 3.84	15.22 ± 6.51	< .0001 [−7.19–−4.63]	< .0001 [−13.38–−9.38]	< .0001 [−7.79–−3.15]
BCVf (D)	0.89 ± 0.16	0.79 ± 0.24	1.56 ± 0.82	< .0001 [0.01–0.19]	< .05 [−0.92–−0.42]	< .0001 [−1.03–−0.51]
SIb(D)	0.01 ± 0.13	0.32 ± 0.24	1.11 ± 0.44	< .0001 [−0.40–−0.22]	< .0001 [−1.24–−0.96]	< .0001 [−0.94–−0.64]
KVb(µm)	8.20 ± 3.27	27.16 ± 7.86	39.31 ± 12.11	< .0001 [−21.68–−16.24]	< .0001 [−34.93–−27.29]	< .0001 [−16.58–−7.72]
BCVb(D)	0.10 ± 0.18	0.68 ± 0.54	1.62 ± 0.98	< .0001 [−0.76–−0.40]	< .0001 [−1.82–−1.22]	< .0001 [−1.28–−0.60]

KVb is the highest point of ectasia on the back surface (µm), KVf is the highest point of ectasia on the front surface (µm), SIb is the Symmetry Index on the back surface curvature map (D), SIf is the Symmetry Index on the front surface curvature map (D).

BCVb = Baiocchi–Calossi–Versaci back, BCVf = Baiocchi–Calossi–Versaci front (D), CI = confidence interval, KVb = Keratoconus Vertex back, KVf = Keratoconus Vertex front, SD = standard deviation, SIb = Symmetry Index back, SIf = Symmetry Index front.

* Student’s *t*-test. Values in square brackets represent the 95% confidence intervals of the mean differences.

### 3.5. HOAs

Total RMS and individual HOAs, including coma, trefoil, and spherical aberrations, increased progressively from controls to Stage 2 KC (Table 3). Notably, coma showed the strongest correlation with disease stage, supporting its role as a functional correlate of structural deformation. Spherical aberration became less negative with progression, consistent with anterior flattening and posterior steepening.

**Table 3:** Group-wise mean, standard deviation, *P* values and and 95% CIs of mean differences for total corneal aberration and epithelial thickness parameters.

	Control (Mean ± SD)	Stage 1 (Mean ± SD)	Stage 2 (Mean ± SD)	*P* value* and CI (Control vs Stage 1)	*P* value* and CI (Control vs Stage 2)	*P* value* and CI (Stage 1 vs Stage 2)
Total RMS (µm)†	0.21 ± 0.12	0.43 ± 0.25	0.84 ± 0.51	< .0001 [−0.31–−0.13]	< .0001 [−0.79–−0.47]	< .0001 [−0.58–−0.24]
Astigmatizm (µm)†	0.08 ± 0.02	1.16 ± 1.06	1,29 ± 0,35	< .0001 [−1.42–−0.74]	< .0001 [−1.32–−1.10]	.46 [−0.48–0.22]
Coma (µm)†	0.14 ± 0.06	0.65 ± 0.20	0.79 ± 0.32	< .0001 [−0.57–−0.45]	< .0001 [−0.75–−0.55]	< .05 [−0.25–−0.03]
Trefoil (µm)†	0.15 ± 0.04	0.28 ± 0.16	0.43 ± 0.12	< .0001 [−0.18–−0.08]	< .0001 [−0.32–−0.24]	< .0001 [−0.21–−0.09]
Spherical (µm)†	−0.18 ± 0.06	−0.11 ± 0.12	−0.04 ± 0.17	< .005 [−0.11–−0.03]	< .0001 [−0.19–−0.09]	< .05 [−0.13–−0.01]
Mean epithelial thickness (µm)	54.2 ± 3.1	51.1 ± 3.4	48.0 ± 4.0	< .0001 [1.61–4.59]	< .0001 [4.62–7.78]	< .0001 [1.48–4.72]
Minimum epithelial thickness (µm)	51.8 ± 2.9	47.5 ± 3.2	43.9 ± 3.8	< .0001 [2.91–5.69]	< .0001 [6.41–9.39]	< .0001 [2.07–5.13]
Maximum epithelial thickness (µm)	58.3 ± 3.5	54.9 ± 3.7	52.1 ± 4.1	< .0001 [1.75–5.05]	< .0001 [4.51–7.89]	< .001 [1.10–4.50]
Epithelial thickness range (µm)	3.9 ± 1.2	7.4 ± 2.3	8.2 ± 2.6	< .0001 [−4.33–−2.67]	< .0001 [−5.17–−3.43]	.04 [−1.87–0.27]

Total RMS: Total root mean square of high-order aberrations in the 4-mm zone (µm).

Astigmatism: Total astigmatic aberration in the 4-mm zone (µm).

Coma: Total coma-like aberration in the 4-mm zone (µm).

Trefoil: Trefoil aberration in the 4-mm zone (µm).

Spherical: Spherical aberration in the 4-mm zone (µm).

CI = confidence interval, RMS = root mean square, SD = standard deviation.

* Student’s *t*-test. Values in square brackets represent the 95% confidence intervals of the mean differences.

† All parameters were obtained from the 4-mm corneal zone.

### 3.6. Quantitative epithelial pattern assessment

Mean ET decreased from 54.2 ± 3.1 µm in controls to 51.1 ± 3.4 µm in Stage 1 and 48.0 ± 4.0 µm in Stage 2 (*P* < .001). Minimum ET showed similar reductions, while maximum ET also decreased, and the ET range (max–min) increased significantly with disease severity (Table 3). These patterns reflect focal thinning over the cone apex and compensatory peripheral thickening, consistent with early structural remodeling. Importantly, quantitative epithelial pattern assessment correlated topographically with posterior surface indices and HOAs, highlighting its complementary role in detecting subtle keratoconic changes.

### 3.7. Receiver operating characteristic (ROC) curve analysis

**Figure 1.**
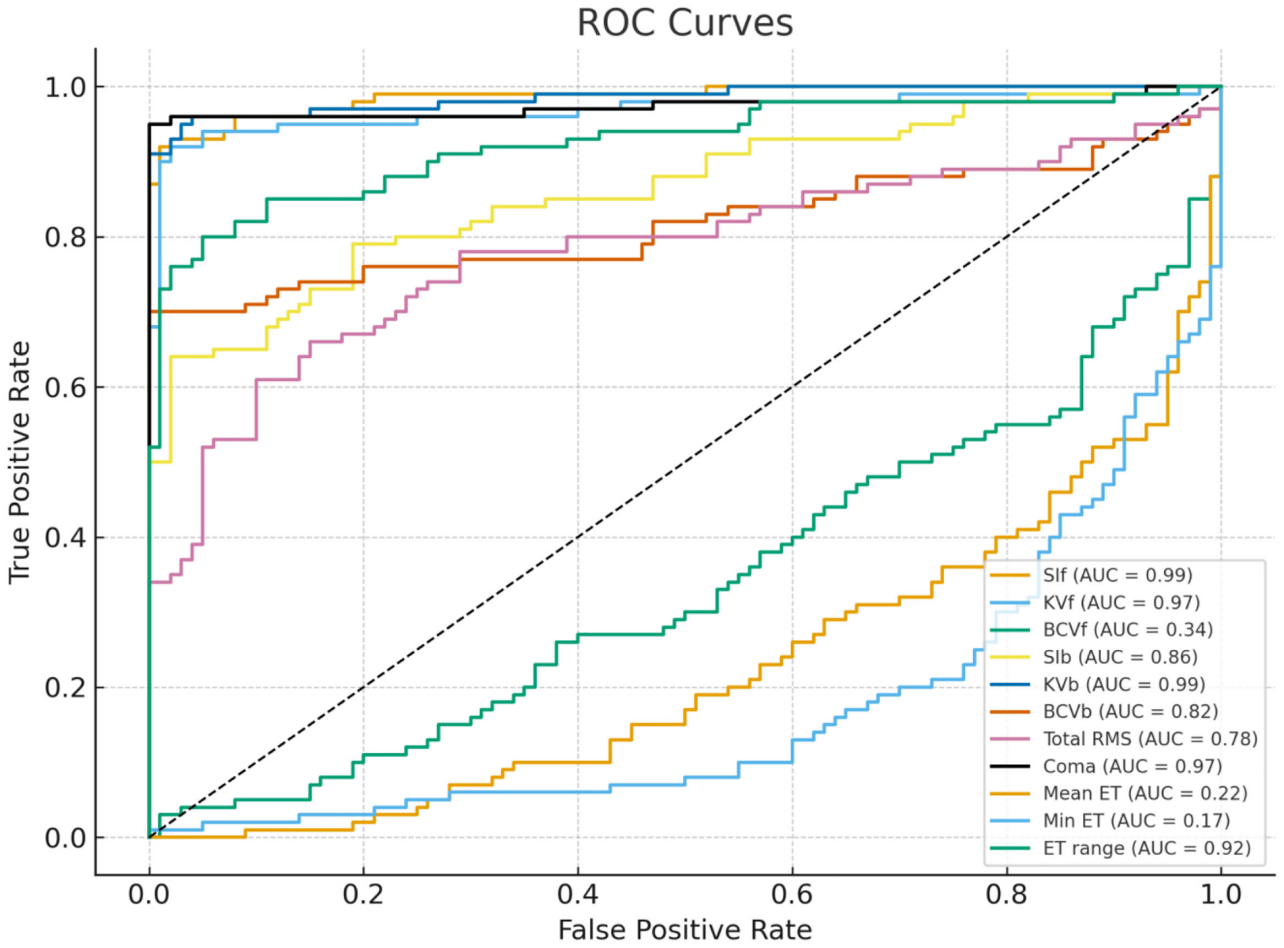
ROC curves for SIf, KVf, BCVf, SIb, KVb, BCVb, total RMS, coma, mean ET, minimum ET, and ET range in distinguishing early-stage keratoconus from normal eyes. The AUC for each parameter is shown in the figure. AUC = area under the curve, BCVb = Baiocchi–Calossi–Versaci back, BCVf = Baiocchi–Calossi–Versaci front, CI = confidence interval, ET = epithelial thickness, KVb = Keratoconus Vertex back, KVf = Keratoconus Vertex front, RMS = root mean square, ROC = receiver operating characteristic, SD = standard deviation, SIb = Symmetry Index back, SIf = Symmetry Index front.

ROC curve analysis was performed to assess the discriminative power of anterior and posterior corneal surface indices, total aberrations, and ET parameters in distinguishing early KC (Stage 1) from normal eyes. The ROC curves demonstrated high diagnostic accuracy for several parameters, particularly the posterior elevation indices (KVb, SIb), front surface asymmetry indices (SIf, KVf), and ET metrics (minimum ET and ET range), all exhibiting high area under the curve values. These findings highlight the strong diagnostic potential of combining tomographic and epithelial mapping parameters for early KC detection (Fig. 1).

### 3.8. Summary of multimodal findings

The integration of Sirius Scheimpflug–Placido tomography with quantitative epithelial pattern assessment provided a comprehensive structural-functional signature of early KC. Progressive anterior and posterior corneal steepening, reduction in pachymetry and Kvol, elevated KC-specific indices, increased HOAs, and localized epithelial thinning collectively distinguished Stage 1 and Stage 2 keratoconic eyes from healthy controls. These findings underscore the synergistic diagnostic utility of posterior surface evaluation, corneal aberrations, and epithelial pattern assessment in early KC detection.

## 4. Discussion

This study provides a comprehensive evaluation of early-stage KC using Sirius Scheimpflug–Placido tomography combined with quantitative ETM. Our results demonstrate that even at the earliest stages, keratoconic eyes exhibit significant and measurable alterations in corneal curvature, pachymetry, posterior corneal elevation, HOAs, and epithelial distribution compared with healthy controls. Anterior curvature parameters, including Kmax and SimK values, showed progressive steepening from controls to Stage 2 eyes, reflecting early biomechanical destabilization of the corneal stroma.^[5,6]^ This anterior steepening was accompanied by thinning of the corneal stroma and reduction in Kvol, suggesting that initial structural compromise is detectable even before advanced clinical signs emerge.^[7,8]^

Posterior corneal indices, particularly SIb, KVb, and BCVb, exhibited high sensitivity in differentiating early KC from normal eyes, consistent with previous reports that posterior elevation changes are often the first detectable sign of ectasia.^[9,10]^ These data underscore the importance of evaluating both corneal surfaces, as reliance on anterior metrics alone may underestimate early ectatic changes. Furthermore, the progressive increase in anterior and posterior KC screening indices (SIf, KVf, BCVf; SIb, KVb, BCVb) aligns with the concept that KC affects the entire corneal architecture, rather than only the anterior surface.^[11,12]^

HOAs, especially vertical coma, increased significantly across disease stages, indicating functional consequences of early structural irregularities.^[13,14]^ Trefoil and spherical aberrations also showed progressive changes, suggesting that even subtle ectatic modifications can measurably affect optical quality. The correlation between posterior surface asymmetry and optical aberrations reinforces the value of combining structural and functional assessments in early detection.^[15,16]^ Our data revealed a progressive increase in total RMS and coma aberration from controls to Stage 2 KC, which is highly consistent with previous optical quality analyses focusing on the functional impact of corneal steepening.^[17,18]^

Quantitative ETM revealed focal thinning over the cone apex and compensatory peripheral thickening. The progressive increase in epithelial range from controls to Stage 2 eyes highlights the sensitivity of this parameter in detecting localized remodeling, which may precede overt topographic changes.^[19,20]^ Unlike conventional epithelial mapping, this approach emphasizes spatial redistribution patterns rather than absolute thickness, providing a more nuanced understanding of epithelial compensation and its relationship with underlying stromal weakness.^[21,22]^ The concordance between posterior elevation, HOAs, and epithelial redistribution suggests that epithelial remodeling reflects an adaptive response to early biomechanical stress, supporting its role as a surrogate marker of corneal instability.^[23,24]^

To address the comparative performance of imaging platforms, it is essential to contextualize our Sirius-based findings within the broader literature. While devices like Pentacam and Galilei are widely used, the Sirius system’s integration of Placido-disk topography with Scheimpflug tomography provides a unique advantage in mapping the anterior surface with superior curvature precision. Previous comparative studies have shown that while Kmax and pachymetry values are generally interchangeable between Sirius and Pentacam, Sirius may offer more sensitive detection of early anterior surface irregularities due to its hybrid nature.^[25]^ Our results confirm that Sirius-derived posterior indices and HOAs provide a diagnostic accuracy comparable to other high-resolution tomographers, particularly when combined with AS-OCT-based epithelial analysis.^[26]^

The integration of curvature, pachymetry, posterior corneal indices, HOAs, and quantitative epithelial patterns creates a reproducible multimodal signature of early KC. Clinically, this framework can guide early intervention with CXL, improve risk stratification for refractive surgery, and allow precise monitoring of disease progression.^[27]^ The superior discriminatory power of posterior indices compared with anterior-only metrics reinforces the need for comprehensive multimodal evaluation, especially in subclinical or borderline cases. This study’s strength lies in its standardized, device-consistent methodology. Utilizing a single Sirius unit for curvature, pachymetry, and corneal indices minimized inter-device variability, while quantitative ETM provided novel insight into early epithelial remodeling.

However, several limitations should be considered. The retrospective design and moderate sample size limit the generalizability of our findings. The absence of direct corneal biomechanical measurements prevents full assessment of the structure-function relationship, and variations in OCT algorithms may affect absolute quantitative epithelial metrics. Future prospective, multicenter studies incorporating biomechanical indices and artificial intelligence (AI) classifiers could refine diagnostic accuracy and predictive modeling for early KC. Recent AI-based analyses combining Zernike polynomial patterns and epithelial topography have demonstrated excellent accuracy in differentiating early KC from normal corneas.^[14,22]^

Overall, our findings support a paradigm shift in KC diagnostics toward comprehensive corneal mapping, where curvature, pachymetry, posterior elevation, HOAs, and epithelial redistribution patterns are collectively analyzed. This multimodal integration allows for earlier detection, improved risk stratification, and individualized management strategies, potentially preventing progression before significant visual impairment occurs. The relationship between epithelial remodeling and corneal biomechanics is particularly relevant. Several reports have suggested that epithelial thinning patterns correspond to localized biomechanical weakness. Although our study did not include direct biomechanical metrics, the concurrent epithelial and posterior surface changes suggest that ETM can serve as a noninvasive surrogate marker for biomechanical instability.

One of the key strengths of our investigation lies in its multimodal, device-consistent approach. By using the same Sirius unit for curvature, pachymetry, and surface indices, and a standardized AS-OCT protocol for epithelial mapping, inter-device variability was minimized. The findings provide evidence that when multiple corneal biomarkers are analyzed in unison, diagnostic confidence markedly improves compared with single-parameter evaluation. This supports the growing consensus that multimodal integration represents the future of KC screening. The clinical implications of our results are substantial. Early multimodal detection facilitates risk stratification for CXL, enabling timely intervention before biomechanical failure occurs. Incorporating ETM and posterior indices into preoperative assessments may prevent iatrogenic ectasia following refractive surgery, particularly in borderline corneas. Furthermore, longitudinal ETM analysis could serve as a monitoring tool for disease progression and CXL efficacy, as epithelial remodeling may reflect stabilization or regression after treatment.

In conclusion, our findings highlight that integrating Scheimpflug–Placido tomography with quantitative ETM, posterior surface indices, and HOAs offers a powerful, noninvasive approach for early KC detection, enhancing diagnostic precision and potentially transforming clinical management strategies.

## 5. Conclusion

This study provides compelling evidence that early-stage KC is not merely a focal anterior surface disorder, but a multifaceted corneal disease characterized by early, coordinated structural and optical changes. By integrating Sirius Scheimpflug–Placido tomography with quantitative ETM, we have identified a robust, reproducible multimodal signature for early ectatic detection. This signature encompasses anterior curvature steepening, posterior elevation, HOAs, and spatially redistributed epithelial patterns. Notably, posterior corneal indices, particularly SIb, KVb, and BCVb, emerged as the most sensitive and specific markers, significantly outperforming traditional anterior metrics.

The clinical synergy of these parameters offers a sophisticated, noninvasive framework that potentially redefines current screening protocols. Unlike conventional topography, our comprehensive assessment provides a more nuanced understanding of how the epithelium adapts to biomechanical stress before overt topographic changes occur. This proactive diagnostic approach facilitates timely intervention with CXL, informs refractive surgery candidacy with higher precision, and allows for the rigorous monitoring of disease stability or progression.

Furthermore, the integration of structural, optical, and epithelial metrics establishes a critical foundation for future predictive modeling using AI and biomechanical analysis. By moving beyond single-modality evaluations toward a comprehensive multimodal paradigm, this study adds a clinically actionable dimension to KC diagnostics. Ultimately, this shift enables a more personalized level of corneal care, potentially preventing significant visual impairment through the earliest possible detection and intervention.

## Acknowledgments

All procedures performed in studies involving human participants were conducted in accordance with the ethical standards of the institutional and/or national research committee and with the 1964 Helsinki Declaration and its later amendments or comparable ethical standards.

## Author contributions

**Conceptualization:** Hande Hüsniye Telek, Huri Sabur.

**Data curation:** Hande Hüsniye Telek, Tugce Horozoglu Ceran, Yaprak Arzu Özdemir.

**Formal analysis:** Hande Hüsniye Telek, Tugce Horozoglu Ceran, Yaprak Arzu Özdemir.

**Investigation:** Hande Hüsniye Telek, Huri Sabur.

**Methodology:** Hande Hüsniye Telek, Huri Sabur, Tugce Horozoglu Ceran, Yaprak Arzu Özdemir.

**Project administration:** Hande Hüsniye Telek.

**Supervision:** Hande Hüsniye Telek, Huri Sabur, Tugce Horozoglu Ceran, Yaprak Arzu Özdemir, Serkan Akkaya.

**Writing – original draft:** Hande Hüsniye Telek.

**Writing – review & editing:** Hande Hüsniye Telek, Huri Sabur, Tugce Horozoglu Ceran, Yaprak Arzu Özdemir, Serkan Akkaya.
